# Prognostic Role of High-Sensitivity Modified Glasgow Prognostic Score for Patients With Operated Oral Cavity Cancer: A Retrospective Study

**DOI:** 10.3389/fonc.2022.825967

**Published:** 2022-02-15

**Authors:** Yao-Te Tsai, Ku-Hao Fang, Cheng-Ming Hsu, Chia-Hsuan Lai, Sheng-Wei Chang, Ethan I. Huang, Ming-Shao Tsai, Geng-He Chang, Chih-Wei Luan

**Affiliations:** ^1^ Department of Otorhinolaryngology-Head and Neck Surgery, Chang Gung Memorial Hospital, Chiayi, Taiwan; ^2^ Department of Otorhinolaryngology-Head and Neck Surgery, Chang Gung Memorial Hospital, Linkou, Taiwan; ^3^ Department of Radiation Oncology, Chang Gung Memorial Hospital, Chiayi, Taiwan; ^4^ Department of Radiology, Chang Gung Memorial Hospital, Chiayi, Taiwan; ^5^ Department of Otorhinolaryngology-Head and Neck Surgery, Lo Sheng Sanatorium and Hospital Ministry of Health and Welfare, Taoyuan, Taiwan

**Keywords:** high-sensitivity modified Glasgow prognostic score, oral cavity squamous cell carcinoma, overall survival, disease-free survival, nomogram

## Abstract

**Aim:**

We probed the prognostic value of the preoperative high-sensitivity modified Glasgow prognostic score (HS-mGPS), neutrophil/lymphocyte ratio (NLR), and platelet/lymphocyte ratio (PLR) for patients with oral cavity squamous cell carcinoma (OSCC) to identify patients with the highest risk of having poor survival outcomes.

**Materials and Methods:**

We executed a retrospective assessment of the records of 303 patients with OSCC who had been subjected to curative surgery between January 2008 and December 2017. The HS-mGPS was categorized using C-reactive protein and albumin thresholds of 3 mg/L and 35 g/L, respectively. Moreover, receiver operating characteristic curve analyses were executed to find out the optimal PLR and NLR cutoffs. We plotted survival curves and compared them through the use of the Kaplan–Meier method and log-rank test, respectively. Through a Cox proportional hazard model, we identified prognostic variables. We also plotted a nomogram comprising the HS-mGPS and clinicopathological factors and assessed its performance with the concordance index.

**Results:**

The PLR and NLR cutoffs were 119.34 and 4.51, respectively. We noted an HS-mGPS of 1−2 to be associated with a shorter median overall survival (OS) and disease-fee survival (DFS) compared with an HS-mGPS of 0. Multivariate analysis revealed that an HS-mGPS of 1−2 and an NLR of ≥4.51 were independent risk factors related to poor OS and DFS. The HS-mGPS appeared to have better prognostic effect than did the PLR and NLR, and the combination of the HS-mGPS and NLR appeared to exhibit optimal discriminative ability for OS prognostication. The nomogram based on the HS-mGPS and NLR yielded accurate OS prediction (concordance index = 0.803).

**Conclusion:**

Our findings suggest that preoperative HS-mGPS is a promising prognostic biomarker of OSCC, and the nomogram comprising the HS-mGPS and NLR provided accurate individualized OSCC survival predictions.

## Introduction

Oral cavity cancer is the commonest malignancy in the head and neck region. Of malignant tumors in the oral cavity, 90% are categorized as oral squamous cell carcinoma (OSCC) (1). The global OSCC incidence is increasing, with over 300,000 cases diagnosed in 2020 (2). For OSCC, the current mainstay of treatment entails curative surgery that is executed along with or without adjuvant therapy. Despite the application of advanced diagnostic modalities and multidisciplinary management, the OSCC prognosis remains unsatisfactory, and approximately 40% of patients experience locoregional recurrence and distant metastasis (3). Therefore, the identification of practical biomarkers for OSCC prognosis would be of clinical value. Studies have reported that several molecular biomarkers related to cancer cell differentiation and proliferation, metastasis, and angiogenesis could be applied to enhance OSCC survival estimations (4). However, the molecular biomarkers and costly laboratory techniques employed may not be widely applicable in clinical practice.

Increasing evidence demonstrates that cancer-related inflammation in the tumor microenvironment has a crucial role in various cancer development stages; such inflammation affects the proliferation and migration of tumor cells, induces angiogenesis and distant metastasis, and diminishes treatment responses to anticancer therapies (5). Furthermore, tumor-specific immunity is important for cancer surveillance and elimination (6). Several hematologic and biochemical immune inflammation indices have prognostic value for patients with OSCC; examples of these indices include serum albumin and C-reactive protein (CRP) levels, the neutrophil/lymphocyte ratio (NLR), and the platelet/lymphocyte ratio (PLR) (7–9). The modified Glasgow prognostic score (mGPS), which incorporates both serum CRP (cutoff: 10 mg/L) and albumin (cutoff: 35 g/L) levels, is an independent overall survival (OS) and cancer-specific survival predictor in OSCC (10). Based on a stricter serum CRP level (3 mg/L) cutoff, the high-sensitivity mGPS (HS-mGPS) has been reported to be a better prognostic indicator than conventional mGPS for various malignancies (11–13), and this superiority was also indicated in a study on head and neck cancer (14). However, the aforementioned study enrolled only 35 (27.1%) patients with OSCC (14), and the authors did not examine several crucial OSCC risk factors in their survival analysis, such as extranodal extension (ENE) (15) and depth of invasion (DOI) (16).

Attempting to fill the aforementioned gap, we executed the current study to probe the prognostic effect of nutrition-inflammation-based indices, including the HS-mGPS, NLR and PLR in patients with OSCC receiving curative surgery. We also investigated whether a prognostic model based on the combination of these indices would be useful for screening out patients with the highest risk of having a poor prognosis. We hypothesized that HS-mGPS and OSCC prognosis would have a significant association given that HS-mGPS reflects both cancer-related inflammation and host nutrition status. A confirmation of our hypothesis would indicate that preoperative HS-mGPS could be used for the early detection of high-risk patients and for treatment optimization, ultimately improving OSCC prognosis.

## Materials and Methods

### Study Design and Populations

We executed the current retrospective single institute study to probe the clinical outcomes of patients who had a new OSCC diagnosis and then underwent primary surgery between January 1, 2007, and December 31, 2018, at our hospital. In total, 336 patients with newly diagnosed OSCC and who satisfied the following inclusion criteria were identified from medical records: (1) having pathologically diagnosed OSCC; (2) undergoing treatment with curative surgery with or without adjuvant therapy; (3) being aged more than 18 years old at time of diagnosis. The exclusion criteria were as follows: (1) having incomplete preoperative laboratory data and follow-up records (n = 11); (2) having a diagnosis of a hematologic or autoimmune disease (n = 2); (3) having a history of an active infectious or inflammatory disorder within 1 month prior to OSCC surgery (n = 2); (4) having a cancer history (n = 8); (5) having metastasis or unresectable disease at diagnosis (n = 7); or (6) having undergone neoadjuvant therapy before curative surgery (n = 3). Ultimately, 303 patients were eligible for inclusion, and they constituted our study population. This study’s protocol adhered to the Declaration of Helsinki and was ratified by our institute’s institutional review board.

### Clinical Data Collection

Medical staff collected relevant medical records from the hospital’s electronic resources and patient charts. Preoperative laboratory data—including serum CRP and albumin levels and absolute neutrophil, lymphocyte, and platelet counts—were retrospectively collected within 2 weeks before operation. As prognostic factors, certain clinicopathological features of each patient were reviewed and evaluated, namely surgical margins, cancer cell differentiation, overall cancer stage, age, perineural invasion (PNI), ENE, sex, and DOI. The pathological tumor, node, metastasis (TNM) stage of all patients was recorded on the basis of the latest staging manual set forth by the American Joint Committee on Cancer (AJCC) (8th Edition, 2018). For defining and documenting the presence of underlying comorbidities, we applied the Charlson Comorbidity Index (CCI) score (17). Through the review of patient interview and clinical data and records, we gleaned data on betel nut chewing, cigarette smoking, and alcohol consumption history. A cigarette smoker was construed as someone who smoked ≥10 cigarettes/day for ≥1 year (18). Moreover, a betel nut chewer was construed as someone who chewed betel nuts ≥2 times a day for ≥1 year. An alcohol drinker was construed as a person who consumed >1 alcoholic beverages per week for >6 months (19). Patients with none, one, or at least two of the aforementioned habits were categorized into corresponding exposure groups.

### Calculation of Hematologic and Biochemical Indexes

During the study period, serum CRP (reference value: <5 mg/L) values as well as albumin (reference value: 35–55 g/L) values were measured through an automated analyzer (Roche Hitachi Cobas 8000, Rotkreuz, Switzerland), and hemoglobin, lymphocyte, platelet, and neutrophil counts were determined through a hematology analyzer (Sysmex SE-9000, Kobe, Japan). Preoperative HS-mGPS of 2, 1, and 0 were assigned to patients identified as having both hypoalbuminemia (<35 g/L) and a high CRP level (>3 mg/L), having either hypoalbuminemia or a high CRP level, and having neither hypoalbuminemia nor a high CRP level, respectively (12). To calculate the NLR, the neutrophil count/lymphocyte count ratio was derived; to calculate the PLR, the platelet count/lymphocyte count ratio was derived.

### Treatment Protocol

Each of the included patients underwent routine preoperative workups that involved lab tests, physical exams, medical history taking, computed tomography (CT) or magnetic resonance imaging (MRI) executed on the head and neck, bone scintigraphy, hepatic ultrasonography, and chest radiography. Furthermore, if metastasis was suspected after such workups, chest or abdomen CT scanning was conducted. All patients underwent curative surgery with concomitant unilateral or bilateral neck dissection. For immediately reconstructing surgical defects, plastic surgeons used pedicle, free, or local flaps. Postoperative adjuvant treatment planning was determined by the consensus of the multidisciplinary tumor board of our hospital. Lin et al. (20) provided the detailed adju4vant treatment guidelines; briefly speaking, those identified as having multiple metastatic lymph nodes, ENE, or positive surgical margins were given adjuvant chemoradiotherapy (CRT), and patients with a solitary metastatic neck lymph node and pathologic T4 disease were administered adjuvant radiotherapy (RT). The intensity-modulated radiotherapy dose per fraction was 2 Gy for 5 days a week, and the total doses were 60−66 and 66 Gy for adjuvant RT and adjuvant CRT, respectively. In accordance with patient preferences and physicians’ judgments, the adjuvant chemotherapy regimen was 100 mg/m^2^ intravenous cisplatin once every three weeks or 40 mg/m^2^ intravenous cisplatin once a week.

### Follow-Up

All patients had routine outpatient follow-up every two months, at 3-month intervals, and at 6-month intervals during the first year, during the second year, and after the second year, respectively. They had head and neck MRI or CT at 6-month intervals for a period of 2 years at 12-month intervals after that. Physical examination and flexible fiberoptic examinations were performed during every follow-up session. We derived OS to be the interval spanning from the surgery date to the date of death from any cause, the date of censoring, or December 31, 2019 (the study’s final follow-up date). We also derived disease-free survival (DFS) to be the interval spanning from the curative surgery date to that of treatment failure (according to clinical evidence such as locoregional recurrence or distant metastasis), that of censoring, or that of death.

### Statistical Analysis

Data normality was investigated using the Kolmogorov–Smirnov test. Numbers and percentages were employed to represent how categorical variables were distributed; medians with interquartile ranges and means with standard deviations were applied to express nonnormally and normally distributed continuous variables, respectively. We established optimal NLR and PLR cutoffs through the execution of receiver operating characteristic (ROC) curve analyses and appraised the equivalent areas under the curves (AUCs). Group data had a nonnormal distribution; accordingly, we executed the χ^2^ test to compare the groups’ clinicopathological features for categorical variables and executed the Mann–Whitney *U* test to compare these features for continuous variables. Survival outcomes were assessed and survival curves were compared through the use of the Kaplan–Meier method and the log-rank test, respectively. After testing Cox’s proportional hazards assumption of the Cox model, we determined risk factors that were related to poor OS and DFS by employing the Cox proportional hazard model and estimated the equivalent hazard ratios (HRs) and corresponding 95% confidence intervals (CIs) through univariate and multivariate analyses. In the univariate analysis, we evaluated potential risk factors by employing the log-rank test; for the multivariate analysis, we employed only those determined to have reached the level of statistical significance (*p* < 0.1) in the univariate analysis.

The likelihood ratio χ^2^ (LRχ^2^) test was applied to examine the predictive homogeneity of the indices and their combination. Through the application of Harrell’s concordance index (C-index), with values ranging from 0.5 to 1.0 (21), we could derive predictive accuracy; moreover, through the application of the Akaike information criterion (AIC) as well as Bayesian information criterion (BIC), we could examine prognostic discriminative ability (22). Overall, low BIC and AIC values and high C-index and LRχ^2^ values were associated with more favorable prognostic discriminative ability. The aforementioned C-index values were thought to represent total chance (C-index = 0.5) and perfect predictability (C-index = 1.0) (23). SPSS 21.0 (SPSS Inc., Chicago, IL, USA) constituted the platform on which the aforementioned analyses were executed. Furthermore, we considered statistical significance to be represented by a two-sided *p* value of <0.05.

On the basis of significant clinicopathological variables of multivariate analysis for OS, a prognostic nomogram was constructed through the ‘rms’ package in R (version 5.1-0; Vanderbilt University, Nashville, Tennessee, USA), with OS at 3 and 5 years used as endpoints (24). To analyze the established nomogram’s OS prediction performance, the C-index was applied for both the proposed nomogram and the traditional TNM system. Additionally, a consistency assessment was conducted between actual survival outcomes and nomogram-predicted OS by constructing calibration plots.

## Results

### Baseline Characteristics

The baseline clinicopathological and demographic characteristics of the enrollees are presented in **Table 1**. Of the 303 enrollees, 274 (90.4%) were men. The patients’ median age was determined to be 57 (range, 31–86) years, and 218 (71.9%) patients were younger than 65 years. The top three primary tumor locations were the tongue (*n* = 120, 39.6%), buccal mucosa (*n* = 99, 32.7%), and gum (*n* = 38, 12.5%). Of the patients, 82.5% (n = 250) were smokers, 79.9% (n = 242) were betel nut chewers, and 65.3% (n = 198) were alcohol consumers. Approximately half of the patients had a stage IV disease (n = 153, 50.5%); 21.1% (n = 64) had a stage I disease, 14.5% had a stage III disease (n = 44), and 13.9% (n = 42) had a stage II disease. PNI was present in 78 (25.7%) patients; in addition, 107 (35.3%) patients were identified as having neck lymph node metastasis that was pathologically confirmed, and 62 (20.5%) patients had ENE. In total, 270 (89.1%) patients had OSCC that was well differentiated to moderately differentiated, and 33 (10.9%) patients had OSCC that was poorly differentiated. After surgery, 146 (48.2%) patients received no adjuvant treatment, 42 (13.9%) patients received adjuvant RT, and 115 (37.9%) received adjuvant CRT.

**Table 1:** Baseline characteristics of enrolled patients with oral cavity squamous cell carcinoma.

Variable	Characteristics
Age (years)	
< 65	218 (71.9%)
≥ 65	85 (28.1%)
Sex	
Men	274 (90.4%)
Women	29 (9.6%)
Primary tumor site	
Tongue	120 (39.6%)
Buccal mucosa	99 (32.7%)
Gum	38 (12.5%)
Retromolar trigone	17 (5.6%)
Mouth floor	12 (4.0%)
Lip	11 (3.6%)
Hard palate	6 (2.0%)
Personal Habits	
Cigarette Smoking	250 (82.5%)
Betel nut chewing	242 (79.9%)
Alcohol consumption	198 (65.3%)
AJCC stage	
I	64 (21.1%)
II	42 (13.9%)
III	44 (14.5%)
IV	153 (50.5%)
T classification	
T1	84 (27.7%)
T2	54 (17.8%)
T3	43 (14.2%)
T4	122 (40.3%)
N classification	
N0	196 (64.7%)
N1	29 (9.6%)
N2	63 (20.8%)
N3	15 (4.9%)
PNI	78 (25.7%)
ENE	62 (20.5%)
Cancer cell differentiation	
W−D/M−D	270 (89.1%)
P−D	33 (10.9%)
Surgical margin	
≥ 5 mm	221 (72.9%)
< 5 mm	82 (27.1%)
DOI ≥ 10 mm	141 (46.5%)
Adjuvant therapy	
Not indicated	146 (48.2%)
RT	42 (13.9%)
CRT	115 (37.9%)
CCI	
0	163 (53.8%)
1	89 (29.4%)
≥ 2	51 (16.8%)
HS-mGPS	
0	157 (51.8%)
1	134 (44.2%)
2	12 (4.0%)
Albumin (g/dL), median (IQR)	45 (42−47)
CRP (mg/L), median (IQR)	3.27 (1.20-11.66)
WBC (x 10^3^/μL), median (IQR)	7.8 (6.3−9.7)
Neutrophil (x 10^3^/μL), median (IQR)	4.9 (3.6−6.4)
Lymphocyte (x 10^3^/μL), median (IQR)	2.1 (1.6−2.6)
Platelet (x 10^3^/μL), median (IQR)	240.2 (192.6−286.1)
NLR, median (IQR)	2.4 (1.7−3.4)
PLR, median (IQR)	113.9 (87.7−153.6)

AJCC, American Joint Committee on Cancer; CCI, Charlson comorbidity index; CRT, chemoradiotherapy; DOI, depth of invasion; ENE, extracapsular nodal extension; HS-mGPS, high-sensitivity modified Glasgow prognostic score; IQR, interquartile range; M−D, moderately differentiated squamous cell carcinoma; NLR, neutrophil/lymphocyte ratio; P−D, poorly differentiated squamous cell carcinoma; PLR, platelet/lymphocyte; PNE, perineural extension; PNI, prognostic nutritional Index; RT, radiotherapy; WBC, white blood cell; W−D, well differentiated squamous cell carcinoma.

### Determination of Biomarker Cutoff Values

HS-mGPSs ranged from 0 to 2: 157 (51.8%), 134 (44.2%), and 12 (4.0%) patients had a score of 0, 1, and 2, respectively (**Table 1**). An HS-mGPS value of ≥1 was determined to be significantly associated with an unfavorable prognosis (25), and only 4.0% of the patients had an HS-mGPS of 2; hence, the patients were categorized as follows for the subsequent analysis: those with an HS-mGPS of 0 (n = 157, 51.8%) and those with a score of 1−2 (n = 146, 48.2%). Additionally, the optimal NLR and PLR cutoffs were 4.51 (*p* = 0.009) and 119.34 (*p* = 0.003), respectively, according to an ROC analysis involving the calculation of AUC and Youden’s J-point for balancing specificity and sensitivity (**Supplementary File 1**).

### Association Between HS-mGPS and Clinicopathological Features

As shown in **Table 2**, patients with an HS-mGPS of 1−2 were more likely to have advanced T and N stages (both *p* < 0.001), a late-stage disease (*p* < 0.001), ENE (*p* < 0.001), a DOI of ≥10 mm (*p* < 0.001), a requirement for adjuvant therapy (*p* < 0.001), and a short median survival time (*p* = 0.014). By contrast, an HS-mGPS of 1−2 had no significant association with sex, age, PNI, tumor cell differentiation, or CCI.

**Table 2:** Clinicopathological variables and Hs-mGPS.

Variable	HS-mGPS		*p-*value
	0, n = 157	1−2, n = 146	
Sex			0.992^a^
Men	142 (90.4%)	132 (90.4%)	
Women	15 (9.6%)	14 (9.6%)	
Age			0.790^a^
<65	114 (72.6%)	104 (71.2%)	
≥65	43 (27.4%)	42 (28.8%)	
Overall stage			<0.001^a^
I−II	74 (47.1%)	32 (21.9%)	
III−IV	83 (52.9%)	114 (78.1%)	
pT classification			<0.001^a^
T1−T2	94 (59.9%)	44 (30.1%)	
T3−T4	63 (40.1%)	102 (69.9%)	
pN classification			<0.001 ^a^
N0	117 (74.5%)	79 (54.1%)	
N1−N3	40 (25.5%)	67 (45.9%)	
PNI			0.154 ^a^
Absent	122 (77.7%)	103 (70.5%)	
Present	35 (22.3%)	43 (29.5%)	
ENE			<0.001^a^
Absent	140 (89.2%)	101 (69.2%)	
Present	17 (10.8%)	45 (30.8%)	
Cell differentiation			0.253^a^
W−D/M−D	143 (91.1%)	127 (87.0%)	
P−D	14 (8.9%)	19 (13.0%)	
Surgical margin			0.028^a^
≥ 5 mm	123 (78.3%)	98 (67.1%)	
< 5 mm	34 (21.7%)	48 (32.9%)	
DOI ≥ 10 mm			<0.001^a^
No	104 (66.2%)	58 (39.7%)	
Yes	53 (33.8%)	88 (60.3%)	
Adjuvant therapy			<0.001^a^
Not indicated	94 (59.9%)	52 (35.6%)	
RT	19 (12.1%)	23 (15.8%)	
CRT	44 (28.0%)	71 (48.6%)	
CCI			0.662^a^
0	88 (56.0%)	75 (51.4%)	
1	45 (28.7%)	44 (30.1%)	
≥ 2	27 (15.3%)	27 (18.5%)	
WBC (X10^3^μl), median (IQR)	7.2 (6.0-8.6)	8.6 (6.9-10.8)	<0.001^b^
Neutrophil (X10^3^μl), median (IQR)	4.3 (3.3-5.7)	5.5 (4.3-7.4)	<0.001^b^
Lymphocyte (X10^3^μl), median (IQR)	2.1 (1.6-2.6)	2.0 (1.6-2.5)	0.502^b^
Platelet (X10^3^μl), median (IQR)	230.2 (190.1-269.9)	248.3 (194.7-309.0)	0.008^b^
Survival (months), median (IQR)	47.6 (28.5-67.7)	37.1 (13.0-65.3)	0.014^b^

CCI, Charlson comorbidity index; CRT, chemoradiotherapy; DOI, depth of invasion; ENE, extranodal extension; HS-mGPS, high-sensitivity modified Glasgow prognostic score; IQR, interquartile range; M−D, moderately differentiated squamous cell carcinoma; P−D, poorly differentiated squamous cell carcinoma; PNI, prognostic nutritional Index; RT, radiotherapy; WBC, white blood cell count; W−D, well differentiated squamous cell carcinoma.

a The Chi-square test.

b The Mann-Whitney U test.

### Correlation Between HS-mGPS and OS

**Figure 1:**
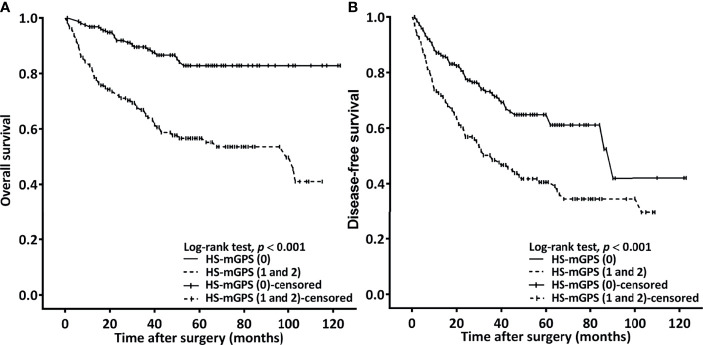
Kaplan–Meier curves plotted to estimate OS **(A)** and DFS **(B)** on the basis of patients’ HS-mGPS.

Through our OS analysis in which the median (range) follow-up period was 40.9 (1.4−122.7) months, we determined an HS-mGPS of 1−2 to be significantly correlated with a shorter median OS (68.1 v.s. 103.2 months, *p* < 0.001) compared with an HS-mGPS of 0 (**Figure 1A**). In our univariate analysis, poor OS indicators were stage IV disease, PNI, poor tumor differentiation, a surgical margin of <5 mm, a need for adjuvant CRT, a CCI of ≥2, an HS-mGPS of 1−2, an NLR of ≥4.51, and a PLR of ≥119.34 (**Table 3**). We performed a multivariate analysis and demonstrated an NLR of ≥4.51 (*p* = 0.002), stage IV disease (*p* = 0.003), poor tumor differentiation (*p* = 0.005), a CCI of ≥2 (*p* = 0.033), an HS-mGPS of 1−2 (*p* < 0.001), and a PLR of ≥119.34 (*p* = 0.046) to constitute independent predictors of poor OS. We identified an HS-mGPS of 1−2 to be correlated with a 2.555 times higher all-cause mortality risk relative to an HS-mGPS of 0. The risk of mortality was 2.339 times higher among patients with a high (≥ 4.51) NLR compared with those with a low (< 4.51) NLR, the mortality risk was 1.208 times higher among patients with a high PLR (≥119.34) compared with those with a low PLR (<119.34).

**Table 3:** Univariate and multivariate analysis of prognostic factors for OS.

Variable	Survival	Univariate analysis		Multivariate analysis	
		HR (95% CI)	*p*-value	HR (95% CI)	*p*-value
Sex					
Women	78.3%	Reference		Reference	
Men	69.0%	1.583 (0.687-3.644)	0.281	0.869 (0.362-2.084)	0.753
Age (years)					
< 65	70.4%	Reference		Reference	
≥ 65	68.6%	1.152 (0.724-1.832)	0.551	1.046 (0.624-1.753)	0.865
Overall stage					
I	92.0%	Reference		Reference	
II	89.6%	0.939 (0.265-3.337)	0.923	0.950 (0.259-3.484)	0.939
III	82.9%	1.790 (0.601-5.329)	0.295	2.114 (0.696-6.420)	0.187
IV	51.6%	6.210 (2.686-14.360)	<0.001	4.191 (1.609-10.918)	0.003
PNI					
Absent	76.0%	Reference		Reference	
Present	52.3%	2.547 (1.641-3.952)	<0.001	1.396 (0.824-2.363)	0.214
Cell differentiation					
W-D/M-D	73.3%	Reference		Reference	
P-D	43.3%	2.855 (1.669-4.885)	<0.001	2.454 (1.320-4.562)	0.005
Surgical margin					
≥ 5 mm	73.2%	Reference		Reference	
< 5mm	61.6%	1.611 (1.028-2.523)	0.037	1.253 (0.771-2.036)	0 0.362
Personal habits					
No exposure	67.3%	Reference			
One exposure	52.0%	1.306 (0.474-3.597)	0.605		0.
Two or all exposure	71.5%	0.957 (0.492-1.862)	0.898		0.
Adjuvant therapy					
Not indicated	81.8%	Reference		Reference	
RT	74.4%	1.524 (0.706-3.287)	0.283	1.151 (0.782-1.816)	0.316
CRT	53.0%	3.621 (2.220-5.906)	<0.001	1.613 (0.811-3.408)	0.471
Tumor subsites					
Tongue	72.8%	Reference			
Buccal mucosa	70.1%	1.166 (0.693-1.960)	0.563		
Other sites	66.2%	1.241 (0.731-2.108)	0.424		
CCI					
0	74.0%	Reference		Reference	
1	71.6%	1.204 (0.717-2.021)	0.484	1.233 (0.701-2.166)	0.467
≥ 2	56.4%	1.943 (1.146-3.294)	0.014	1.899 (1.054-3.420)	0.033
HS-mGPS					
0	82.8%	Reference		Reference	
1−2	56.6%	3.651 (2.224-5.993)	<0.001	2.555 (1.524-4.283)	<0.001
NLR					
< 4.51	76.4%	Reference		Reference	
≥ 4.51	24.8%	4.212 (2.621-6.770)	<0.001	2.339 (1.365-4.007)	0.002
PLR					
< 119.34	78.1%	Reference		Reference	
≥ 119.34	59.1%	2.203 (1.422-3.414)	<0.001	1.208 (1.019-2.105)	0.046

CCI, Charlson Comorbidity Index; CI, confidence interval; CRT, chemoradiotherapy; HR, Hazard ratio; HS-mGPS, high-sensitivity modified Glasgow prognostic score; M-D, moderately differentiated squamous cell carcinoma; NLR, neutrophil/lymphocyte ratio; OS, overall survival; OSCC, oral cavity squamous cell carcinoma; P-D, poorly differentiated squamous cell carcinoma; PLR, platelet/lymphocyte ratio; PNI, perineural invasion; RT, radiotherapy; W-D, well differentiated squamous cell carcinoma.

### Correlation Between HS-mGPS and DFS

An HS-mGPS of 1−2 was noted to be correlated with a significantly shorter median DFS relative to an HS-mGPS of 0 (36.6 vs. 90.5 months, *p* < 0.001; **Figure 1B**). The correlation between clinicopathological variables and DFS is presented in **Table 4**. In our univariate analysis, we determined an NLR of ≥4.51, poor tumor differentiation, stage IV disease, need for adjuvant CRT, an HS-mGPS of 1 or 2, and a PLR of ≥119.34 to exhibit a significant correlation with poor DFS. We demonstrated through a multivariate analysis that an NLR of ≥4.51 (*p* = 0.013), stage IV disease (*p* = 0.004), poor tumor differentiation (*p* = 0.010), an HS-mGPS of 1−2 (*p* = 0.005) constituted independent risk factors for poor DFS. However, our results did not indicate a PLR of ≥119.34 to constitute an independent risk factor for unfavorable DFS.

**Table 4:** Univariate and multivariate analysis of prognostic factors for DFS.

Variable	Survival	Univariate analysis		Multivariate analysis	
		HR (95% CI)	*p*-value	HR (95% CI)	*p*-value
Sex					
Women	68.6%	Reference		Reference	
Men	50.8%	1.452 (0.778-2.710)	0.241	1.052 (0.552-2.004)	0.878
Age (years)					
< 65	51.9%	Reference		Reference	
≥ 65	54.7%	0.889 (0.607-1.302)	0.544	0.868 (0.588-1.281)	0.475
Overall stage					
I	67.5%	Reference		Reference	
II	73.6%	0.682 (0.330-1.407)	0.300	0.694 (0.334-1.444)	0.329
III	63.8%	0.895 (0.457-1.752)	0.745	1.054 (0.528-2.105)	0.882
IV	37.2%	2.204 (1.380-3.521)	0.001	2.312 (1.299-4.115)	0.004
PNI					
Absent	55.9%	Reference			
Present	43.1%	1.392 (0.959-2.021)	0.082		
Cell differentiation					
W-D/M-D	55.9%	Reference		Reference	
P-D	34.7%	1.968 (1.232-3.143)	0.005	1.921 (1.169-3.155)	0.010
Surgical margin					
≥ 5 mm	55.9%	Reference		Reference	
< 5mm	44.5%	1.415 (0.989-2.024)	0.058	1.190 (0.821-1.726)	0.358
Personal habits					
No exposure	63.4%	Reference			
One exposure	39.1%	1.443 (0.598-3.485)	0. 0.415		0.
Two or all exposure	52.1%	1.386 (0.780-2.464)	0. 0.266		0.
Adjuvant therapy					
Not indicated	59.3%	Reference		Reference	
RT	59.3%	0.968 (0.548-1.711)	0.912	0.918 (0.569-1.882)	0.713
CRT	41.6%	1.748 (1.221-2.503)	0.002	1.351 (0.804-2.050)	0.078
Tumor subsites					
Tongue	59.2%	Reference			
Buccal mucosa	50.5%	1.150 (0.760-1.741)	0.508		
Other sites	45.3%	1.478 (0.912-2.225)	0.112		
CCI					
0	52.0%	Reference			
1	58.2%	0.829 (0.550-1.249)	0.369		
≥ 2	46.4%	1.151 (0.740-1.791)	0.532		
HS-mGPS					
0	64.8%	Reference		Reference	
1−2	40.5%	2.038 (1.438-2.888)	<0.001	1.687 (1.168-2.438)	0.005
NLR					
< 4.51	57.8%	Reference		Reference	
≥ 4.51	18.7%	2.500 (1.653-3.781)	<0.001	1.787 (1.131-2.825)	0.013
PLR					
< 119.34	61.3%	Reference		Reference	
≥ 119.34	41.4%	1.766 (1.259-2.479)	0.001	1.256 (0.863-1.829)	0.234

CCI, Charlson Comorbidity Index; CI, confidence interval; CRT, chemoradiotherapy; DFS, disease-free survival; HR, Hazard ratio; HS-mGPS, high-sensitivity modified Glasgow prognostic score; M-D, moderately differentiated squamous cell carcinoma; NLR, neutrophil/lymphocyte ratio; OSCC, oral cavity squamous cell carcinoma; P-D, poorly differentiated squamous cell carcinoma; PLR, platelet/lymphocyte ratio; PNI, perineural invasion; RT, radiotherapy; W-D, well differentiated squamous cell carcinoma.

### Prognostic Efficacy Estimation

**Table 5** shows results derived from comparing the prognostic effect of the indices included, namely the HS-mGPS, NLR, and PLR. Among these indices, the HS-mGPS had the highest LRχ^2^ and C-index scores and the lowest AIC and BIC scores, suggesting its superior prognostic value for determining OS in our study setting. We further investigated the prognostic effect of the combinations of indices by adding the NLR and PLR, separately, to the HS-mGPS; the results demonstrated that the combination of the HS-mGPS and NLR had the highest predictive accuracy (higher C-index), prognostic stratification performance (lower AIC and BIC values), and predictive homogeneity (higher LRχ^2^ score).

**Table 5:** Comparison of the prognostic efficacy for overall survival.

Indexes	LR _X_2 test	AIC	BIC	C-index
HS-mGPS	33.18	649.24	650.26	0.66
NLR	22.60	670.40	671.44	0.61
PLR	11.57	692.45	693.52	0.60
Combinations				
HS-mGPS	–	649.24	650.26	0.66
HS-mGPS + NLR	7.67	928.36	929.83	0.71
HS-mGPS + PLR	7.49	951.38	952.88	0.70
HS-mGPS + NLR + PLR	1.84	1230.47	1232.41	0.71

AIC, Akaike information criterion; BIC, Bayesian information criterion; C-index, concordance index; HS-mGPS, high-sensitivity modified Glasgow prognostic score; LR test, likelihood ratio test; NLR, neutrophil/lymphocyte ratio; PLR, platelet/lymphocyte ratio.

**Figure 2:**
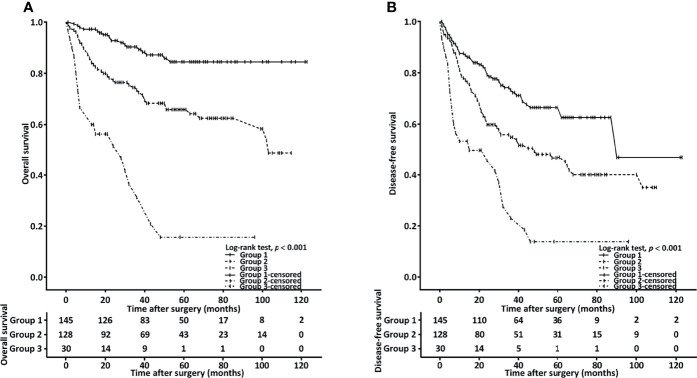
Combined effect of HS-mGPS and NLR on OS **(A)** and DFS **(B)** determined using the Kaplan–Meier method. Group 1, patients with a low NLR (< 4.51) and HS-mGPS of 0; group 2, patients with either an NLR of ≥ 4.51 or HS-mGPS of 1−2; group 3, patients with a high NLR (≥ 4.51) and HS-mGPS of 1−2.

To identify patients with the highest treatment failure risk, we stratified the patients into the following groups: group 1 (n = 145, 47.9%), comprising those with low NLR (< 4.51) and an HS-mGPS of 0; group 2 (n = 128, 42.2%), comprising those with either an NLR of ≥4.51 or an HS-mGPS of 1−2; group 3 (n =30, 9.9%), comprising those with a high NLR (≥ 4.51) and an HS-mGPS of 1−2. On the basis of Kaplan–Meier curves, we probed the OS (**Figure 2A**) and DFS (**Figure 2B**) of the patients in these groups. In groups 1, 2, and 3, the median OS periods were determined to be >103.6, >53.1, and 28.6 (95% CI: 8.6–48.8) months, respectively, and the median DFS periods were derived to be 90.6 (95% CI: 58.2–122.1), 48.4 (95% CI: 19.5–77.6), and 15.3 (95% CI: 4.6–39.2) months, respectively. We noted the groups to differ significantly with respect to OS and DFS, according to the results of a log-rank test (both *p* < 0.001, **Figure 2**). In the multivariate Cox regression analysis, we noted group 3 to be independently associated with the poorest OS (HR = 6.544, 95% CI: 3.253–12.765, p < 0.001, **Table 6**).

**Table 6:** Univariate and multivariate analysis for OS.

Variable	Survival	Univariate analysis		Multivariate analysis	
		HR (95% CI)	*p-*value	HR (95% CI)	*p-*value
Sex					
Women	78.3%	Reference		Reference	
Men	69.0%	1.583 (0.687-3.644)	0.281	0.891 (0.374-2.127)	0.796
Age (years)					
< 65	70.4%	Reference		Reference	
≥ 65	68.6%	1.152 (0.724-1.832)	0.551	1.062 (0.634-1.767)	0.819
Overall stage					
I	92.0%	Reference		Reference	
II	89.6%	0.939 (0.265-3.337)	0.923	0.947 (0.257-3.484)	0.935
III	82.9%	1.790 (0.601-5.329)	0.295	2.102 (0.692-6.388)	0.191
IV	51.6%	6.210 (2.686-14.360)	<0.001	4.243 (1.628-11.057)	0.003
PNI					
Absent	76.0%	Reference		Reference	
Present	52.3%	2.547 (1.641-3.952)	<0.001	1.414 (0.830-2.412)	0.203
Cell differentiation					
W-D/M-D	73.3%	Reference		Reference	
P-D	43.3%	2.855 (1.669-4.885)	<0.001	2.450 (1.315-4.566)	0.005
Surgical margin					
≥ 5 mm	73.2%	Reference		Reference	
< 5mm	61.6%	1.611 (1.028-2.523)	0.037	1.282 (0.788-2.087)	0 0.317
Personal habits					
No exposure	67.3%	Reference			
One exposure	52.0%	1.306 (0.474-3.597)	0. 0.605		0.
Two or all exposure	71.5%	0.957 (0.492-1.862)	0. 0.898		0.
Adjuvant therapy					
Not indicated	81.8%	Reference		Reference	
RT	74.4%	1.524 (0.706-3.287)	0.283	1.156 (0.784-1.818)	0.318
CRT	53.1%	3.621 (2.220-5.906)	<0.001	1.617 (0.815-3.410)	0.472
Tumor subsites					
Tongue	72.8%	Reference			
Buccal mucosa	70.1%	1.166 (0.693-1.960)	0.563		
Other sites	66.2%	1.241 (0.731-2.108)	0.424		
CCI					
0	74.0%	Reference		Reference	
1	71.6%	1.204 (0.717-2.021)	0.484	1.240 (0.706-2.179)	0.455
≥2	56.4%	1.943 (1.146-3.294)	0.014	1.921 (1.068-3.457)	0.029
NLR and Hs-mGPS					
Group1	85.0%	Reference		Reference	
Group2	65.3%	3.210 (1.835-5.617)	<0.001	2.448 (1.367-4.406)	0.003
Group3	15.6%	11.226 (5.878-21.437)	<0.001	6.544 (3.253-12.765)	<0.001

CCI, Charlson Comorbidity Index; CI, confidence interval; CRT, chemoradiotherapy; HR, Hazard ratio; HS-mGPS, high-sensitivity modified Glasgow prognostic score; M-D, moderately differentiated squamous cell carcinoma; NLR, neutrophil/lymphocyte ratio; OS, overall survival; OSCC, oral cavity squamous cell carcinoma; PNI, perineural invasion; P-D, poorly differentiated squamous cell carcinoma; RT, radiotherapy; W-D, well differentiated squamous cell carcinoma.

### Prognostic Nomogram

**Figure 3:**
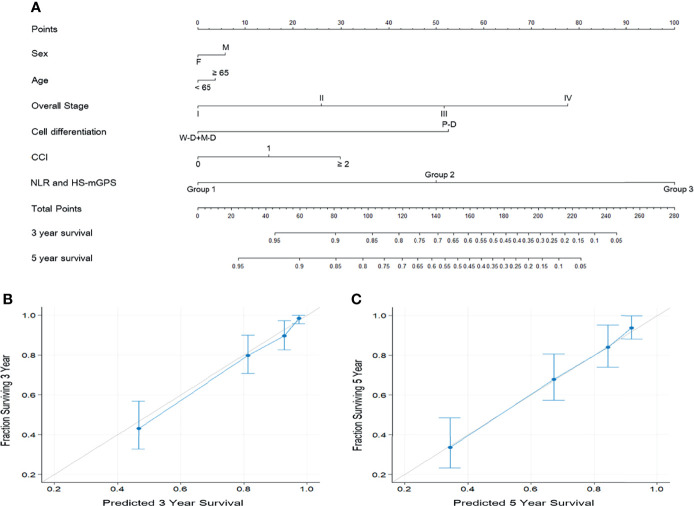
Prognostic nomogram predicting the OS of patients with OSCC **(A)**. The nomogram was constructed and interpreted as follows: A vertical line was drawn from each variable to the uppermost horizontal axis marked as points. The points where the vertical line crosses the axis represent the degree of risk a variable contributes. The points from all variables were added to obtain the summarized points, and a vertical line was drawn from the points on the axis marked as total points to the below axes marked as 3-year survival and 5-year survival to obtain the 3- and 5-year OS predictions of the nomogram. Calibration plots for **(B)** 3-year OS and **(C)** 5-year OS predictions revealed high agreement between the OS predictions of the nomogram and actual survival outcomes. The 45° light gray line represents the ideal survival prediction, and the blue line indicates the predicted outcomes. Blue dots with bars represent the performance and 95% CI of the nomogram when applied to cohorts that survived.

To provide an accurate estimation of OS based on our study findings, we constructed a prognostic nomogram that comprised the aforementioned groups’ model, overall stage, sex, cancer cell differentiation, and age (**Figure 3A**). As the nomogram shows, the model combined HS-mGPS and NLR had the strongest effect on OS, followed by TNM stage and cancer cell differentiation. Regarding OS prediction, the derived C-index for the established nomogram was 0.803, exceeding that of the nomogram consisting of TNM staging alone (C-index = 0.695, **Supplementary File 2**). Additionally, the calibration plots of the nomogram for 3-year (**Figure 3B**) and 5-year (**Figure 3C**) OS estimations revealed the predicted and actual survival outcomes to have a satisfactory level of consistency between them.

## Discussion

Effective prognostic markers for better treatment planning and patient stratification related to OSCC are pressing requirements. Evidence demonstrates that systemic inflammation and host nutrition status play key roles in cancer angiogenesis and progression (26, 27), and several inflammation- and nutrition-based indices, such as NLR and PLR, have been proposed as prognostic biomarkers for patients with OSCC (28, 29). Hanai et al. reported that the HS-mGPS but not mGPS was an independent prognostic marker for head and neck cancer; the HS-mGPS may help identify patients with cachexia sensitively in those with an early-stage disease or good performance status, highlighting the prognostic impact of optimal nutritional support (14). However, only 35 participants with oral cavity cancer were enrolled in their study, and the absence of robust evidence for the clinical use of the HS-mGPS for patients with OSCC precludes the formulation of clear recommendations. According to a literature review, our current study represents the first examination of the HS-mGPS’s prognostic value for patients with OSCC. We observed that an HS-mGPS of 1−2 was associated with a late disease stage, a need for adjuvant therapy, advanced T and N stages, ENE, a DOI of ≥10 mm, and a short median survival. Additionally, our multivariate analysis revealed an HS-mGPS of 1−2 to constitute an independent risk factor for poor DFS and OS. We compared the prognostic efficacy of the HS-mGPS, NLR, and PLR as well as that of their combination. When we applied the LRχ^2^ value, C-index, and AIC and BIC values, the HS-mGPS appeared to have the best prognostic effects, and the HS-mGPS combined with the NLR revealed the optimal prognostic discriminative ability. To identify patients with the highest treatment failure risk, we grouped our patients according to the HS-mGPS (0 or 1−2) and NLR (< or ≥ 4.51). We revealed that patients with a high NLR (≥ 4.51) and an HS-mGPS of 1−2 had the poorest OS, suggesting that an HS-mGPS of 1−2 and NLR of ≥4.51 may indicate some synergism that contributes to a poor OSCC prognosis. Currently, TNM staging system is the most widely used classification system of OSCC for treatment planning and prognosis estimation. The prognostic nomogram that incorporated the TNM staging system, group model, and clinicopathological variables yielded more favorable results compared with the nomogram that was based on TNM staging alone (C-index: 0.803 vs 0.695); therefore, the established nomogram may provide more accurate patient stratification and aid personalized treatment planning. Our study results confirm the HS-mGPS’s prognostic value for patients with OSCC, suggesting the consideration of host nutrition and inflammatory factors as rational adjuncts to the staging of OSCC. Given that the mainstay of treatment for OSCC is ablative surgery, patients who are estimated to have poor prognosis from the proposed nomogram might require more aggressive adjuvant management and close follow up, which warrants further investigation.

The HS-mGPS integrated with both systemic inflammation (CRP) and host nutrition status (albumin level) is a biochemical index established by Proctor et al., who modified the serum CRP threshold from 10 mg/L (conventional mGPS) to 3 mg/L to increase the index’s prognostic importance (30). One of the main advantages of the HS-mGPS is that it can be easily obtained from laboratory tests, and its prognostic superiority over GPS and mGPS has been reported in studies involving various cancers (12, 13, 31). Our study results reveal that the HS-mGPS had better prognostic value than did the NLR and PLR, which is in line with the results of studies examining patients with soft tissue sarcoma (32); this result may be explained by the fast reactivity of CRP and the interaction of host nutrition status and anti-tumor immune response in determining HS-mGPS. Nevertheless, the underlying mechanism connecting the HS-mGPS and OSCC prognosis remained uncertain, and it may be explained as follows. Studies have indicated that OSCC increases the secretion of interleukin 6 and 8 (33), which potentially results in CRP synthesis within the liver and may cause autocrine tumor growth factor activity to induce head and neck cancer progression (34). Extensive tumor invasion and necrosis may also positively upregulate systemic inflammation, and the resultant elevated CRP level is linked to a poor OSCC prognosis (35). A chronic malnutrition indicator, hypoalbuminemia is associated with a poor head and neck cancer prognosis (36). Hwang et al. revealed serum CRP and albumin levels to be negatively associated (37), and this is possibly explained by the decreased hepatic synthesis of albumin due to increased systemic inflammation (38). Cancer-related systemic inflammation and a poor nutrition status may be reflected by a high HS-mGPS, and the resultant sarcopenia has a synergistic effect with systemic inflammation, conferring a poorer prognosis on patients with advanced OSCC (39). The aforementioned studies have suggested the possible mechanism through which a high HS-mGPS negatively influences DFS and OS within OSCC. Further large-scale prospective research should be executed to validate our findings and clarify the underlying mechanism of the association of the HS-mGPS with OSCC prognosis.

PLR and NLR were identified by our study as constituting independent prognostic factors for OS. As suggested by Hasegawa et al., who conducted a study on patients with primary OSCC, preoperative NLR elevation is an independent predictor of poor disease-specific survival as well as poor OS in such patients; this finding is in line with our results (40). However, unlike the study by Chen et al. (41), our study did not reveal an independent role of PLR in predicting DFS. This discrepancy may be explained by the difference between the PLR cutoff values and AJCC manual editions used in the mentioned studies. Lymphocytes, neutrophils, and platelets are involved in carcinogenesis not only in systemic circulation but also in the tumor microenvironment. Studies have reported that platelets downregulate natural killer cell activity through transforming growth factor beta secretion, modulate tumor angiogenesis through the vascular endothelial growth factor pathway, and help circulating tumor cells to adhere to the microvascular endothelium, thereby encouraging tumor cell progression and metastasis (42, 43). Lymphocytes are essential to host antitumor immunity through their induction of direct tumor cell cytotoxicity and cytokine secretion, such as tumor necrosis factor alpha and interferon gamma (44). Therefore, decreased lymphocyte counts may reflect impaired immune surveillance and favorable conditions for tumor progression, which eventually indicated a poor cancer-related prognosis (45). Neutrophils promote tumor-related angiogenesis through cytokine and chemokine secretion and release nitric oxide as well as reactive oxygen species, engendering downregulation of T-cell function and consequently enhancing cancer cell invasion, proliferation, and metastasis (46). A high NLR and PLR may reflect increased cancer-related inflammation and a weakened antitumor immune response, which are associated with a poor prognosis related to various cancers (47). Nevertheless, no consensus has been reached on optimal NLR and PLR cutoffs because tumor location, cancer stage, and age distribution may contribute to variations in cutoffs, rendering it difficult to verify the validity of such cutoffs in different studies. By contrast, the cutoffs of serum CRP and albumin levels used in HS-mGPS calculations have been well validated with regard to various tumors (11–13). Therefore, HS-mGPS could be useful for OSCC patient stratification, surpassing the NLR and PLR in terms of clinical applicability and validity.

Studies have reported that the HS-mGPS is significantly correlated with tumor aggression. For example, patients with prostate cancer and an HS-mGPS of ≥1 had significantly higher prostate-specific antigen and testosterone levels than did those with an HS-mGPS of <1 (48). Another study also identified a significant association between a high HS-mGPS and a larger tumor size and higher grade of soft tissue sarcoma (31). A study on hypopharyngeal cancer detected no significant correlation of the GPS with patients’ clinicopathological characteristics (49). By contrast, we revealed an HS-mGPS of 1−2 to exhibit a significant correlation with advanced-stage disease, more advanced T and N stages, ENE, and a DOI of ≥10 mm, suggesting that the HS-mGPS may have high sensitivity for predicting head and neck cancer aggression. The assumed mechanism underlying these correlations are as follows: (1) A substantial tumor burden, such as greater tumor volume and nodal metastasis, may occur along with high cancer-related systemic inflammation, thus possibly increasing CRP (50) and interleukin (33) levels and thereby engendering a high HS-mGPS (2). Higher cancer-related inflammation increases the depletion of serum albumin and impairs the synthesis of albumin within the liver (51), contributing to a high HS-mGPS. All the aforementioned studies have provided potential insight into how the HS-mGPS is associated with the OSCC aggression, which warrants further verification and investigation.

Our study’s strength is the use of a relatively large data set of patients with OSCC who received curative surgery as a primary treatment. After identifying the prognostic value of the HS-mGPS, we demonstrated that the combination of the biochemical (HS-mGPS) and hematological (NLR) indices enabled comprehensive and refined OSCC prognostication. The established nomogram based on this combined model confirmed the clinical applicability of the HS-mGPS and NLR and provided accurate individualized survival prediction. However, the study was not without limitations. First, the retrospective and single-institution design may have introduced bias. To minimize potential bias, we enrolled a relatively large number of patients with OSCC who were all treated with curative surgery. Second, we did not use an independent data set to confirm our derived results, meaning external validity remained unconfirmed. Furthermore, the HS-mGPS, NLR, and PLR were all measured before surgery. Future research should explore the changes in these indices in response to curative treatment over time and examine how these changes are associated with OSCC prognosis.

## Conclusions

Our study indicated the HS-mGPS to be a promising prognostic biomarker for patients with OSCC who have undergone curative surgery. The HS-mGPS could be considered in clinical practice due to the high availability, reproducibility, and low cost of this biomarker-related approach to patient prognosis estimations. The established nomogram comprised the combination of HS-mGPS and NLR and confirmed the clinical applicability of the combined model with accurate individualized survival estimations. Further large-scale prospective research is necessary to validate our findings.

## Data Availability Statement

The raw data supporting the conclusions of this article will be made available by the authors, without undue reservation.

## Ethics Statement

Chang Gung Memorial Hospital’s Institutional Review Board ratified our executed study (board approval number: 202100555B0C601). The patients/participants provided their written informed consent to participate in this study.

## Author Contributions

Y-TT and C-WL conceived, designed, and supervised the study. K-HF, C-MH, and C-HL collected the data of patients and followed up. S-WC and EH analyzed the data. M-ST and G-HC provided technical assistance with the data analysis. Y-TT and C-WL wrote the manuscript. All authors contributed to the article and approved the submitted version.

## Funding

This work was supported by a grant (CMRPG6L0231) from Chang Gung Memorial Hospital, Taiwan.

## Conflict of Interest

The authors declare that the research was conducted in the absence of any commercial or financial relationships that could be construed as a potential conflict of interest.

## Publisher’s Note

All claims expressed in this article are solely those of the authors and do not necessarily represent those of their affiliated organizations, or those of the publisher, the editors and the reviewers. Any product that may be evaluated in this article, or claim that may be made by its manufacturer, is not guaranteed or endorsed by the publisher.

## Supplementary Material

Supplementary File 1 Receiver operating characteristic curve and AUC analyses of the NLR and PLR. AUC, area under the curve; NLR, neutrophil/lymphocyte ratio; PLR, platelet/lymphocyte ratio.

Supplementary File 2 Kaplan–Meier estimates of overall survival **(A)** and disease-free survival **(B)** according to the cancer stages.
