# Supplementary figures and images for: Prognostic Role of High-Sensitivity Modified Glasgow Prognostic Score for Patients With Operated Oral Cavity Cancer: A Retrospective Study
Source: Front Oncol. 2022 Feb 15;12:825967. doi: 10.3389/fonc.2022.825967 (PMC8886616; doi:10.3389/fonc.2022.825967)

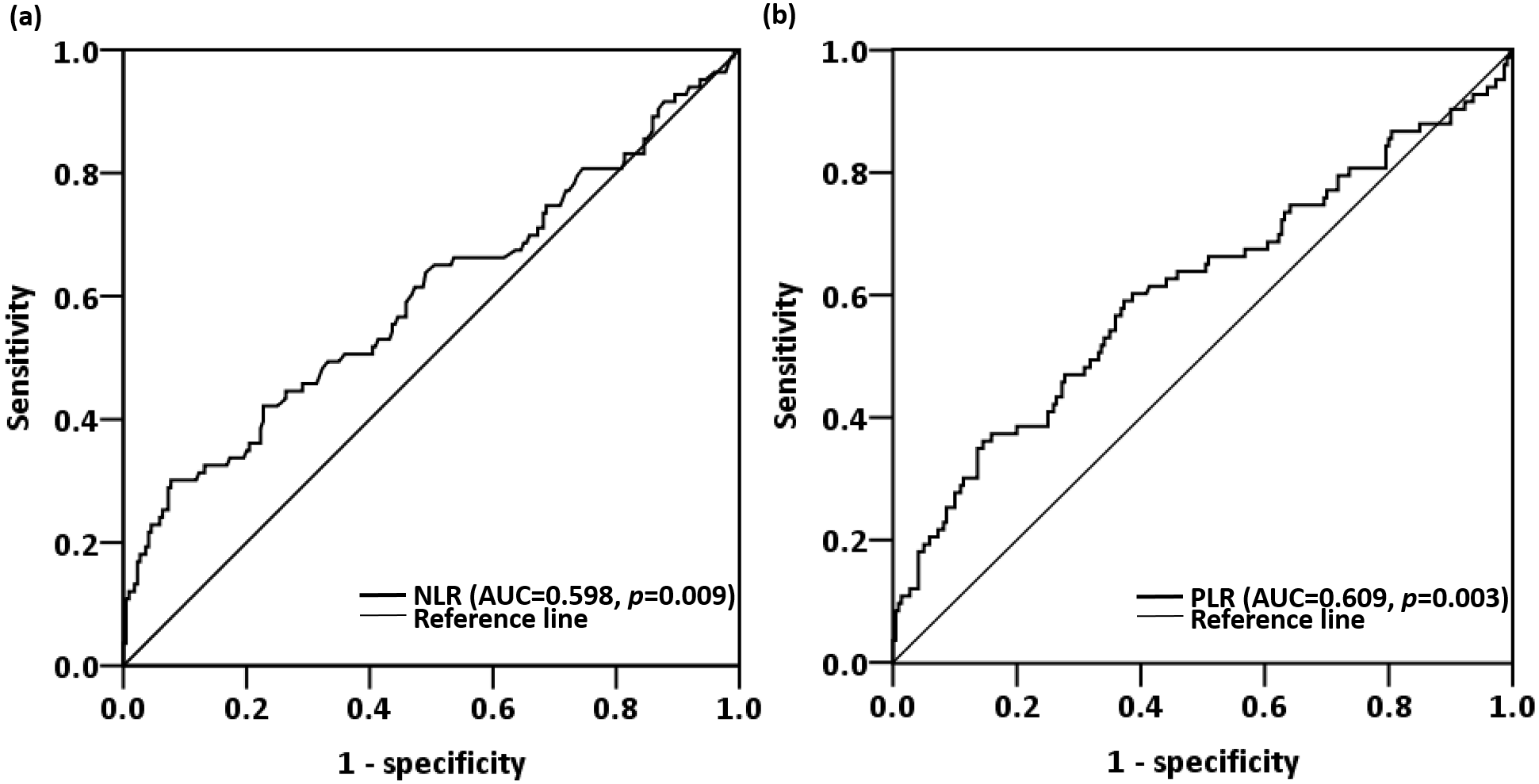

Supplement: Supplementary File 1 — Receiver operating characteristic curve and AUC analyses of the NLR and PLR. AUC, area under the curve; NLR, neutrophil/lymphocyte ratio; PLR, platelet/lymphocyte ratio. [file Image_1.tiff]

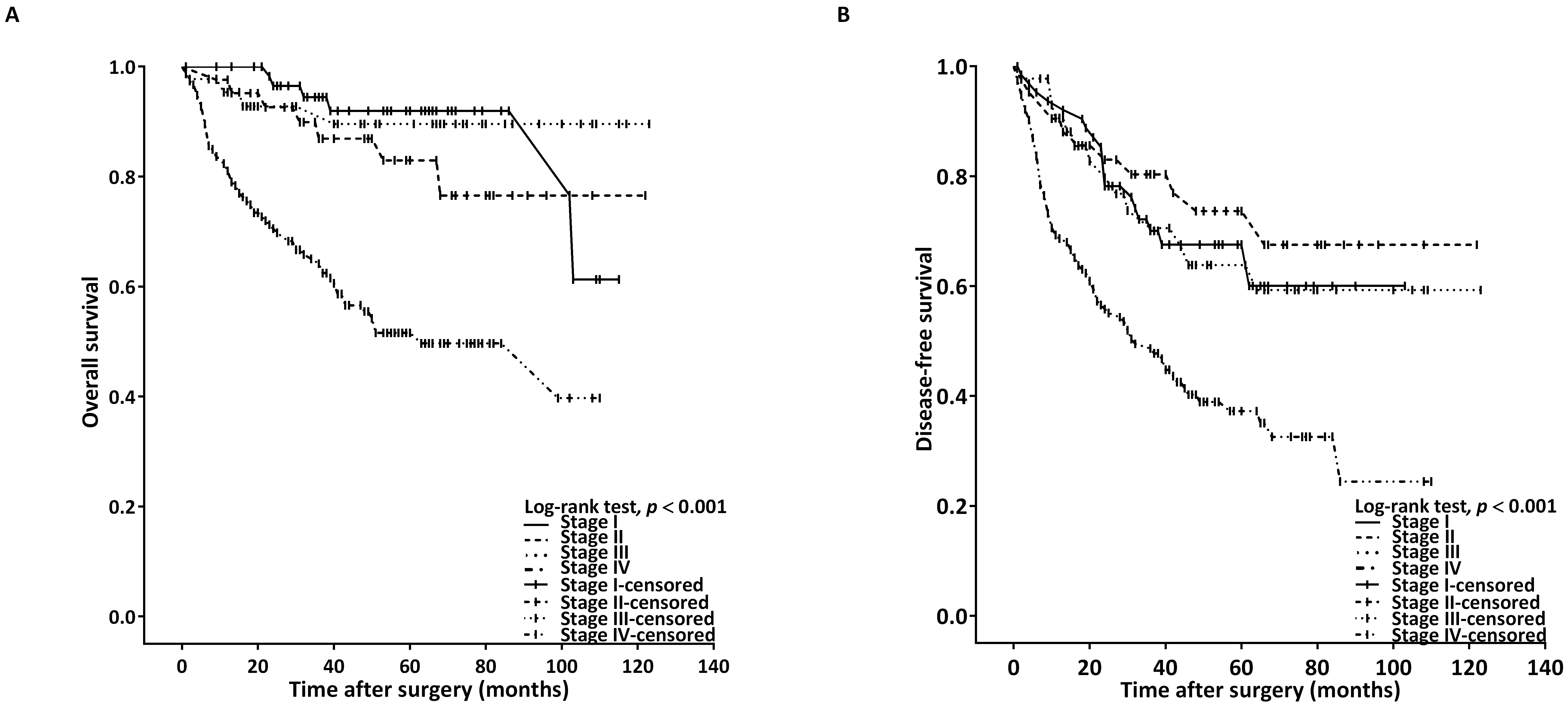

Supplement: Supplementary File 2 — Kaplan–Meier estimates of overall survival (A) and disease-free survival (B) according to the cancer stages. [file Image_2.tiff]
